# Challenges and approaches in tracking and analyzing movement of ground-dwelling insects

**DOI:** 10.1186/s40462-026-00637-x

**Published:** 2026-03-04

**Authors:** Jana Růžičková, Zoltán Elek

**Affiliations:** 1 HUN-REN-ELTE-MTM Integrative Ecology Research Group, Biological Institute, ELTE Eötvös Loránd University, Pázmány Péter sétány 1/C, Budapest, 1117 Hungary; 2 Department of Systematic Zoology and Ecology, Biological Institute, ELTE Eötvös Loránd University, Pázmány Péter sétány 1/C, Budapest, 1117 Hungary; 3 Department of Biostatistics, University of Veterinary Medicine Budapest, István utca 2, Budapest, 1078 Hungary; 4 HUN-REN-DE Anthropocene Ecology Research Group, University of Debrecen, Egyetem tér 1, Debrecen, 4032 Hungary

**Keywords:** Arthropods, Capture-mark-recapture, Non-lethal methods, Telemetry, Tracking, Trajectories

## Abstract

The movement ecology of ground-dwelling insects is essential for understanding their behavior and population dynamics. These insects, especially flightless beetles and orthopterans, exhibit cryptic lifestyles and fine-scale movements that pose unique challenges to tracking and recording their movement paths under natural field conditions. In this review, we evaluate existing tracking methods and propose an analytical framework for studying ground-dwelling insect movement, emphasizing their applications, limitations, and future directions. Traditional methods such as capture-mark-recapture and direct observation have been increasingly complemented by technologies like radio telemetry and harmonic radar, which can provide high-resolution movement trajectories. Despite these advances, many movement studies remain descriptive, focusing on basic metrics such as distances and turning angles without addressing underlying behavioral or ecological processes. Innovative analytical tools such as hidden Markov models and step-selection functions, well-established in vertebrate movement ecology, are rarely adapted to insect movement studies. This limits our ability to explore potential links between the movement of ground-dwelling insects and behavioral patterns, habitat features, or environmental changes. By framing movement analyses along a descriptive–mechanistic–predictive continuum, we highlight the need for more integrative, process-based approaches to improve our understanding of how these insects respond to habitat fragmentation and climate change. Since no single method fits all scenarios, we hope this review guides entomologists in selecting appropriate methods and analytical tools for studying ground-dwelling insects across diverse ecological contexts.

**Supplementary Information:**

The online version contains supplementary material available at 10.1186/s40462-026-00637-x.

## Background

Traditional entomological sampling often relies on lethal methods, where insects are trapped and killed for scientific purposes. Their widespread use has been strongly shaped by the perception that insects are abundant and resilient, essentially “running the world” [1, 2], and by regulatory frameworks that offer far stronger protection to vertebrates than to invertebrates. As a result, insects are still largely excluded from formal ethical standards, partly because they are not considered sentient in the same way as vertebrates and partly because applying species-level welfare rules to such diverse groups is logistically difficult [3, 4]. Still, lethal sampling methods have contributed substantially to our ecological and taxonomic knowledge, and although they are sometimes complemented by non-lethal or minimally invasive options such as in situ observations, live pitfall trapping, or presence–absence surveys (e.g., [5, 6]), they remain common practice. Growing ethical and conservation concerns, however, are shifting attention toward non-destructive techniques [4]. Beyond ethics, lethal sampling has a fundamental biological limitation: it prevents repeated observations of the same individual. Such repeated measurements are essential for quantifying movement patterns, including distance, direction, and timing between movement steps. This makes non-lethal methods central to studying individual movement because they allow tracking of the same insects over time without harming them.

Studying the movement of free-ranging insects under natural conditions has a long history, from direct observation of marked individuals or their footprints to the use of advanced technological tools [7], including remote sensing [8, 9]. Despite this tradition, insects remain the focus of only a small fraction of movement ecology studies: about 11% overall [10], and just 7% of studies published between 2009 and 2018 [11]. Yet, understanding insect movement is fundamental to a range of ecological processes. At the individual level, movement determines access to mates, resources, oviposition sites, and the ability to avoid predators or unfavorable conditions, ultimately affecting fitness. At broader scales, individual movements, particularly dispersal and habitat use, aggregate to shape population dynamics, gene flow, metapopulation stability, and species distributions [12]. Capturing fine-scale movement patterns is therefore essential, as it allows researchers to quantify the mechanisms underlying these patterns, linking individual behavior to broader ecological outcomes. Moreover, in response to various disturbances, insects often exhibit immediate behavioral adjustments, typically expressed as altered movement activity to seek out more suitable habitats. Thus, understanding when, where, and why insects move across various landscape configurations is crucial not only for interpreting ecological mechanisms but also for detecting early warning signals of environmental change and, ultimately, for making predictions [5, 13]. This is especially important given recent declines in insect populations and shifts in their phenology [14–17].

In this review, we provide a systematic overview of non-lethal techniques for monitoring the movement of ground-dwelling insects, especially flightless beetles, orthopterans, and phasmatodeans. We focus on ground-dwellers because of their relatively cryptic lifestyle. Unlike flying insects, whose conspicuous flights can often be followed with the naked eye or binoculars (e.g., [18, 19]), ground-dwellers exhibit slower and finer-scale movements, often hidden within leaf litter, grass tufts, or bushes. Moreover, their movements reflect a variety of behaviors, such as foraging, dispersal, predator avoidance, and search for suitable habitat. These patterns may vary in extent and duration, which affects what can be observed or measured. Capturing these different movement patterns, therefore, requires selecting the best available tracking methods and analytical approaches. The slow and cryptic nature of ground-dwellers also limits the feasibility of remote tracking commonly used for pollinators or migratory species (e.g., [9, 20–22]), therefore, traditional field-based monitoring often remains the most practical option. In addition, limited dispersal abilities make ground-dwellers especially sensitive to habitat alterations [5, 23, 24].

**Fig. 1:**
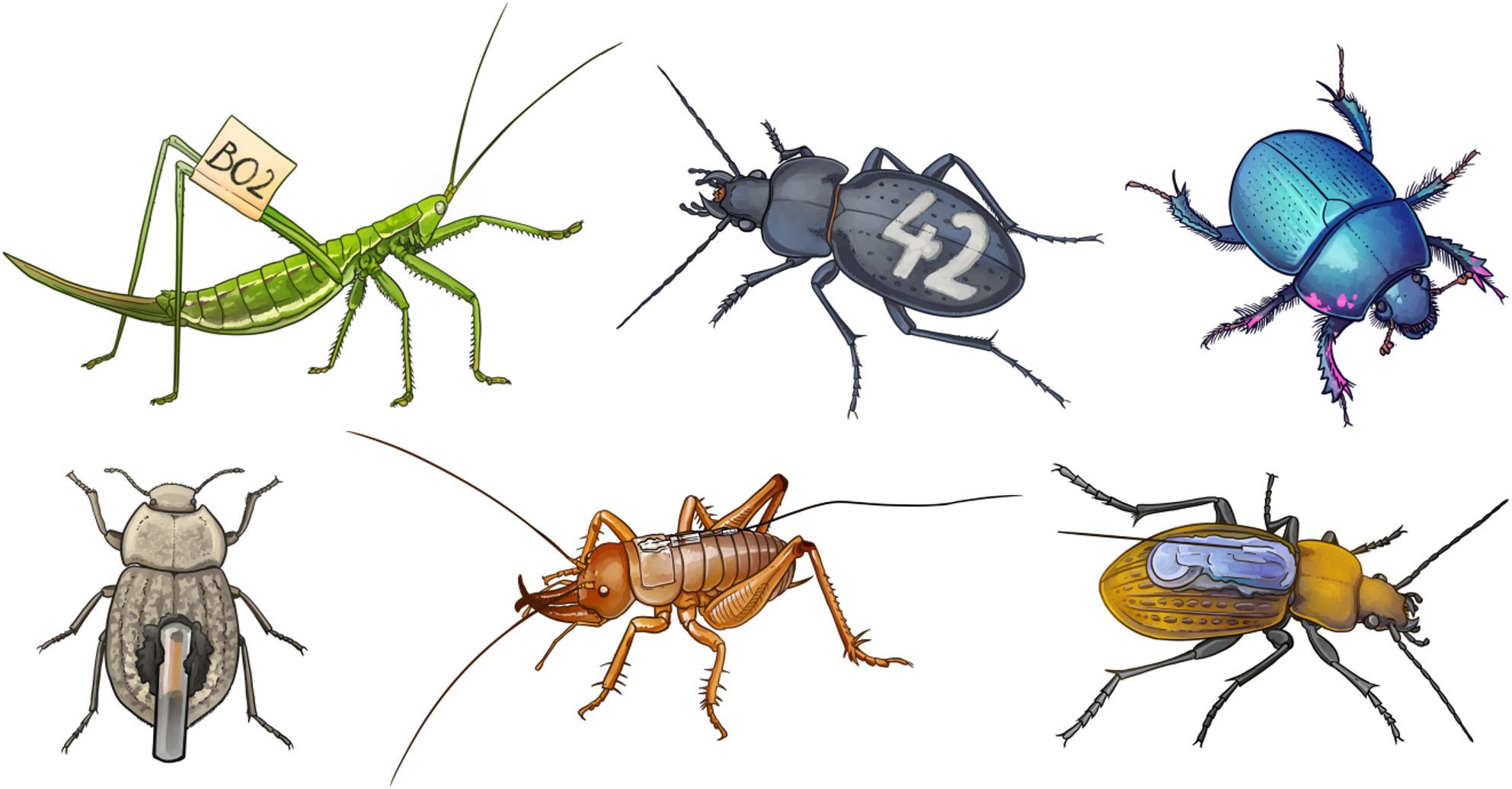
Various methods for marking and tagging ground-dwelling insects. From top left to bottom right: the bush cricket *Saga pedo* with a fluorescent tag on the right hind femur [26]; the ground beetle *Carabus hungaricus* with an engraved number on the elytra [27]; the dung beetle *Anoplotrupes stercorosus* marked with flecks of fluorescent powder on the legs and pronotum [28]; the tenebrionid beetle *Asida sericea* equipped with an RFID tag [29]; the Mercury Islands tusked weta *Motuweta isolata* with a harmonic radar transponder attached to the pronotum [30]; and the ground beetle *Carabus ullrichii* fitted with a VHF radio transmitter [31]. Images are not to scale, while the body size of *A. sericea* is approximately 12 mm, and *S. pedo* can reach up to 105 mm from head to ovipositor tip

Although fine-scale movement measurements remain challenging, various methods now allow researchers to mark and follow ground-dwelling insects under natural conditions across different temporal and spatial scales (see Fig. 1 for examples). To provide a broad and representative overview of the field, we conducted a rapid systematic search of the Web of Science database, focusing on studies of ground-dwelling insect movement ecology. We first summarize commonly used tracking methods, highlighting their strengths and limitations in terms of data resolution, quantity, and quality, and then address how raw movement data can be analyzed to extract meaningful ecological insights. Despite the growing availability of advanced statistical tools, entomology has been slow to adopt these practices compared to vertebrate-focused fields such as ornithology and mammalogy (e.g., [11, 13]). This is likely because insect movement data are often sparse, irregular, or limited to presence-absence observations, and because research has historically emphasized taxonomic and abundance-based approaches rather than behavior (e.g., [6, 22, 25]). To structure the literature and clarify research objectives, we classified studies by tracking methods and along a simple continuum of their research goals, from descriptive through mechanistic to predictive. Descriptive studies quantify basic movement metrics, mechanistic studies link movement patterns to internal or external drivers, and predictive studies aim to forecast movement under changing environmental conditions [13]. In this review, we aim to build a critical and contemporary synthesis on tracking methods and analytical approaches for studying the movement of ground-dwelling insects to provide clear guidelines for methodological choices and advance the conceptual understanding of their movement across ecological contexts.

## Literature search and selection

**Fig. 2:**
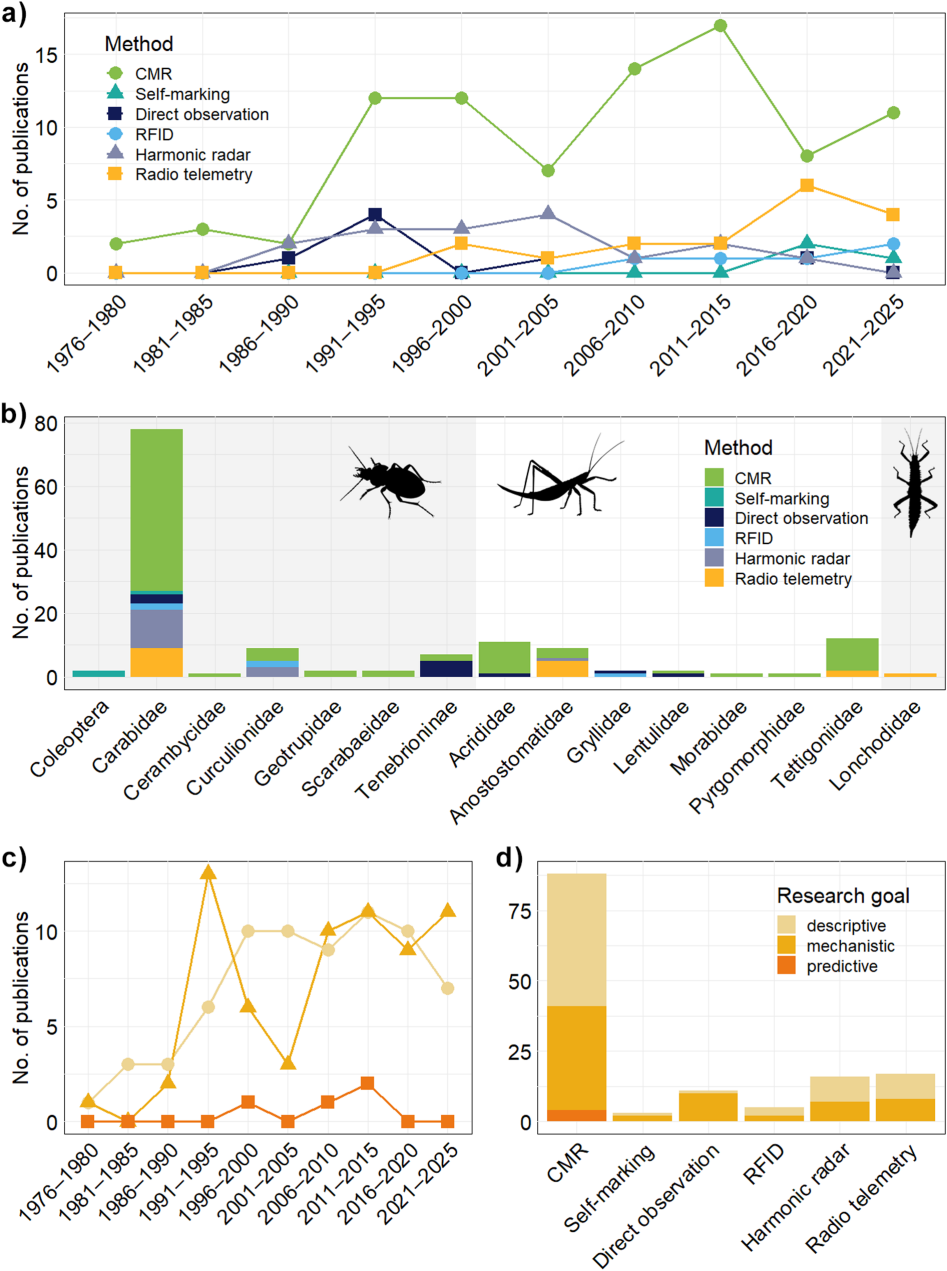
Five decades of movement ecology of ground-dwelling insects. (**a**) Number of published records per five-year interval for each tracking method, i.e., capture-mark-recapture (CMR), self-marking, direct observation, radio-frequency identification (RFID), harmonic radar, and radio telemetry. (**b**) Distribution of tracking methods across insect taxa and families, with silhouettes illustrating beetles, orthopterans, and phasmatodeans. (**c**) Temporal trends in research goals (descriptive, mechanistic, and predictive; color legend matching panel d) across five decades of research. (**d**) Proportion of research goals associated with each tracking method

This review is based on a systematic search of the Web of Science Core Collection conducted on 27 October 2025, covering the time period between 1975 and 2025. We included primary research articles, proceedings papers (as the journal’s special issue), reviews, and book chapters from the disciplines Ecology, Entomology, Zoology, and Biodiversity Conservation. The search string attempted to cover three concept groups: (1) movement-related terms, capturing studies on spatial behaviour and activity patterns; (2) taxonomic scope, focusing on ground-dwelling insects and other terrestrial arthropods, including commonly studied model taxa; and (3) tracking and observation methods, selecting studies that recorded individual movement using field-based techniques. The full search string is provided in the Supplementary Material S1. The search initially retrieved 1,379 records, which were screened for relevance based on titles and abstracts. We also screened the reference lists of all relevant studies to identify additional publications. We excluded movement studies on flying or aquatic insects, laboratory-only research, theoretical papers, and articles written in languages other than English. The final literature database comprised 132 studies listed in the Supplementary Material S2. From each study, we extracted the focal taxa, the tracking method used (capture–mark–recapture, self-marking, direct observation, radio-frequency identification, harmonic radar, and radio telemetry), and the primary research goal, defined as the analytical aim of the study. We classified these aims along a descriptive–mechanistic–predictive continuum, reflecting increasing levels of ecological inference and analytical complexity. Descriptive goals summarize basic movement metrics, mechanistic goals relate movement patterns to underlying processes (such as home range, dispersal, behavior, or habitat use), and predictive goals seek to anticipate movement across space and time under changing environmental conditions. Since several studies applied more than one tracking method, this extraction yielded 141 methodological records in total, spanning five decades of research (Fig. 2a). One record [32] employed a now-abandoned technique, but it identified the key movement patterns of ground-dwelling insects and was therefore retained in the database. The dataset was dominated by studies on ground beetles (Carabidae; 79 records) and orthopterans from the families Tettigoniidae (12 records) and Acrididae (11 records, Fig. 2b), while other families of beetles and orthopterans were less represented and there was only one study on the movement of phasmatodeans [33].

## Current state of movement research in ground-dwelling insects

### Fine-scale movement paths and basic characteristics

Ground-dwelling insects generally move more continuously than flying species, whose movement is often determined by stopovers [34]. This continuity allows relatively reliable estimates of potential movement speed and distance [5, 35]. Despite continuous movement, insect trajectories are recorded as a series of discrete points, called “fixes”, each representing an individual’s steady position at a specific time [34]. Successive fixes define *step lengths* (the distance between consecutive positions) and *turning angles* (the bearing between steps; [36, 37]). Fixes also provide the basis for quantifying basic movement characteristics, including daily dispersal, net displacement (straight-line distance between the first and last fix), total path length (the sum of all step lengths), movement speed (step length divided by the time interval), path tortuosity, and activity rate (the rate between fixes with and without movement). These reliable metrics describe how individuals move through space and provide a basis for linking individual behavioral decisions to external factors such as habitat use and landscape structure. Segmenting trajectory into fixes also allows for detailed movement analyses [38], although periods of inactivity of ground-dwelling insects, sometimes lasting several days, could complicate behavioral predictions [39]. More fixes generally improve spatial and temporal resolution, but precision depends on the model species, study design, and tracking method ([35]; Table 1). The scale of each fix can vary widely, for instance, direct observations can provide step lengths of just a few centimeters when recorded every few seconds or minutes [40, 41], whereas radio telemetry studies typically yield longer steps, measured in meters when fixes are taken every few hours [31, 39], and may extend to hundreds of meters if observations are limited to once per day [42].

**Table 1:** An overview of the main sampling methods used in ground-dwelling insect movement ecology, including key advantages, limitations, and suitability for descriptive, mechanistic, and predictive research goals. CMR stands for capture-mark-recapture, and RFID for radio-frequency identification

Method	Pros	Cons	Suitable for research goal
CMR	Well-established method; suitable for many species; large sample sizes; allows long-term monitoring; cost-effective	Labor-intensive; low recapture rates; limited fine-scale movement data; dependent on trap placement and surface activity	Descriptive (basic movement characteristics); Mechanistic (home range; dispersal; habitat use); Predicitve (long-term dispersal)
Self-marking	Minimal handling, low disturbance, simultaneous marking of multiple individuals and species, large sample sizes; cost-effective	Low temporal resolution; no individual ID, short persistence of markings, limited to crossing predefined zones	Descriptive (directions); Mechanistic (limited, short-term habitat use)
Direct observation	Detailed behavioral and movement data; minimal equipment required; cost-effective	Short observation periods; limited simultaneous viewing; limited to daylight activity; small sample sizes.	Descriptive (basic movement characteristics); Mechanistic (home range; dispersal; behavior; microhabitat use)
RFID	Extremely lightweight; suitable for small insects; allows individual identification	Short detection range, limited applicability for free-ranging individuals; labor-intensive; cost	Descriptive (basic movement characteristics); Mechanistic (limited, dispersal, habitat use)
Harmonic radar	Lightweight, high-resolution movement data; allows long-term monitoring	No individual ID; limited simultaneous tracking; limited detection range; impaired by dense vegetation, moisture, and terrain; labor-intensive, cost	Descriptive (basic movement characteristics); Mechanistic (home range; dispersal; behavior; habitat use); Predicitve (long-term studies)
Radio telemetry	High precision; high-resolution movement data; simultaneous tracking of multiple individuals	Restricted to large species; single-use transmitters; labor-intensive, cost	Descriptive (basic movement characteristics); Mechanistic (home range; dispersal; behavior; habitat use); Predicitve (long-term studies)

Recording accurate fixes can be challenging because daily dispersal often falls within the error range of standard instruments. Handheld GPS devices, commonly used for larger or flying species, typically lack the precision needed at fine scales, where step lengths of two meters or less can be strongly affected by GPS error [43]. An alternative is the distance–bearing method, where each fix is derived from the previous one using distance and compass azimuth [34, 44]. Distances can be obtained manually with a measuring tape or using electronic distance meters, which minimize measurement error [35]. Compass bearings can also be measured with smartphones equipped with magnetic and gravity sensors, which can correct for magnetic declination [45]. This method is highly precise, but labor-intensive, as it requires numerous manual measurements in the field.

Movement data should ideally include both spatial (coordinates) and temporal information [34]. Most R packages for movement analyses are designed for GPS-based tracking data of vertebrates, where positions are given as longitude and latitude. For insects, manually measured distances and azimuths must first be converted into long/lat coordinates [43] or projected into a planar system such as the Universal Transverse Mercator (UTM). Unlike geographic systems, which use degrees as basic units and spherical projection, UTM projects positions on a flat, two-dimensional grid in meters. This makes the calculation of step lengths and turning angles straightforward using Euclidean geometry [46]. UTM projection improves precision for fine-scale insect trajectories, although it is limited to latitudes between 84°N and 80°S and can complicate analyses if movements cross zone boundaries.

### Sampling methods

Most methods for studying the movement of ground-dwelling insects rely on some form of marking to distinguish individuals. Marking options vary widely, from painted labels to radio tags glued to the insect’s cuticle (Fig. 1), but share essential features. They should be durable, easy to apply, clearly visible, and cause minimal or no impact on insect behavior [47]. Nevertheless, especially with methods using tagging, behavioral effects related to tag size, weight, or placement remain insufficiently understood [48, 49]. The choice of marking technique also depends heavily on the species’ size and ecology, and methods suitable for one species may be entirely ineffective for another [47].

Beyond marking-based techniques, various insect tracks and signs, especially footprints, can offer an alternative way to record movement without capturing or tagging individuals. Here, movement paths are reconstructed retrospectively from footprints preserved in soft substrates (e.g., sand, soft bare soil surface) or on tracking plates or cards, where individuals walk over pigment and leave their footprints on the canvas [50]. Despite their potential, footprint-based observations have been used mainly for presence–absence surveys (e.g., [51, 52]) rather than for quantifying the movement of ground-dwelling insects, likely due to limitations on small areas of suitable substrate and the lack of options for individual identification. We therefore treat this method as complementary and do not consider it further among the core sampling methods.

In this chapter, we review six commonly used methods for tracking ground-dwelling insects identified through our systematic search: capture–mark–recapture, self-marking, direct observation, radio-frequency identification, harmonic radar, and radio telemetry. For each sampling method, we provide a brief introduction, explain the basic concept, describe typical study designs, summarize the type and resolution of the movement data obtained, and discuss the main advantages and limitations. At the end of each section, we also highlight key applications. This structure may help researchers to compare these sampling methods and their suitability for different research goals (see Table 1 for an overview).

#### Capture-mark-recapture (CMR)

CMR is the most widely used and traditional method for studying ground-dwelling insect movement (Fig. 2a). More than half of the methodological records (88 records; 62.4%) in our review relied on CMR, reflecting its long history and versatility. The method involves capturing individuals, marking them with unique identifiers, such as UV paint, engraved numbers, or reflective tags, and releasing them to be recaptured later. Each recapture represents a fix representing the insect’s location at a specific time. In ground-dwelling insect studies, typical CMR designs deploy unbaited pitfall traps arranged in grids, transects, or circles to maximize spatial coverage [27, 53–56]. Alternatively, individuals can be released into large enclosures and recaptured along perimeter barriers [57–59]. Flightless orthopterans are often captured using sweep nets in low vegetation or shrubs [60]. CMR provides fixes with step lengths typically on the scale of meters, and the minimum interval between recaptures is usually one day, while in practice it can extend to several days or even weeks, depending on surface activity, trap placement, and search effort. However, the total duration of the experiment can last several years, especially if engraving is used for marking individuals, making CMR one of the best methods for long-term movement studies. It can address a broad spectrum of research goals, from estimating basic movement characteristics (e.g., daily dispersal, e.g., [61, 62]), to quantifying dispersal distances (e.g., [63, 64]), home-range size (e.g., [65]), habitat edge permeability (e.g., [66]), and overall habitat use (e.g., [54, 59]), and even predicting long-term dispersal (e.g., [60]). The main advantages of CMR are its cost-effectiveness, applicability across species, and suitability for monitoring multiple individuals simultaneously. Limitations include labor-intensive fieldwork, especially when recaptures require nocturnal searches with flashlights or UV lamps [26, 61, 67]. In addition, due to low recapture rates, it typically shows only a subset of an individual’s movement path, limiting the reconstruction of fine-scale trajectories or behavioral patterns. Taken together, CMR is well-suited for cost-effective, long-term studies on habitat use and edge permeability. However, it provides only coarse movement paths and very limited behavioral patterns, capturing mainly broad processes such as dispersal and habitat fidelity.

#### Self-marking

Self-marking technique is a relatively new and rare method for studying ground-dwelling insect movement, with only three studies (2.1%) identified in the last decade (Fig. 2a). This method is particularly useful when researchers aim to assess movement directions of multiple individuals and species simultaneously with minimal handling. The basic principle relies on insects marking themselves by contacting naturally occurring substances (e.g., pollen) or intentionally applied materials (e.g., coloured powders, chemical solutions) in their environment [47, 68]. For ground-dwelling insects, UV-fluorescent pigments are particularly effective, as they can be applied in bands directly on the soil. Individuals crossing the band acquire the powder and can later be collected and inspected under UV light, providing a record of their movement across the marked area [28]. The experimental designs involve strategically placing powder bands at the edges of habitats, in corridors, or between small patches of habitat. Researchers can collect insects from adjacent areas after applying the powder to determine whether individuals have crossed the marked zone. Because the method does not require individual handling during marking, it can simultaneously track multiple species from different taxonomic groups (Fig. 2b). Self-marking provides edge-crossing movement data rather than continuous trajectories, making it ideal in small-scale or short-term contexts [69]. Nevertheless, temporal resolution is low, and the exact timing of crossing events is unknown. Moreover, individual identification is not possible, and the fluorescent powder often wears off within days. Overall, self-marking is suitable for short-term, small-scale studies on spillover from one habitat to another or edge crossing, allowing simultaneous tracking of multiple species. However, it does not allow individual identification, and provides neither details about movement trajectories nor behavior.

#### Direct observations

Direct observation allows researchers to capture very fine-scale movement trajectories and behavioral patterns of ground-dwelling insects directly in the field. This method was first applied in the late 1980s, and we identified 11 records (7.8%) to date using it (Fig. 2a). Individuals are visually followed from a short distance (1–2 m), and their positions are recorded at regular intervals [41]. Sequentially numbered sticks or flags may be placed in the ground to mark each location, although placement is sometimes delayed by one or two steps to minimize disturbance [35, 40, 70]. Each recorded location constitutes a fix, allowing reconstruction of step lengths and turning angles. Direct observation offers high temporal resolution, with fixes recorded every few seconds or minutes, while spatial resolution depends on the observer’s attention. The method captures detailed movement paths and behavioral patterns, making it ideal for very fine-scale trajectory reconstruction and subsequent analyses (e.g., [40, 71]). It is particularly valuable for examining basic movement characteristics and mechanistic research goals, such as behavioral patterns, home-range estimation, and microhabitat use [41, 72], but it is generally not appropriate for long-term or predictive movement analyses due to its short duration. In addition, it is labor-intensive, typically allows only short tracking periods for one individual at a time, is mostly limited to daylight activity, and the presence of an observer may influence insect behavior. To sum up, direct observation is ideal for recording fine-scale movement trajectories, behavior, and microhabitat use in relatively small, open areas. Nevertheless, its use is limited to short-term, daylight observations, and it generally cannot provide insight into long-term or movement predictions.

#### Radio-frequency identification (RFID)

RFID was first applied in ground-dwelling insect studies in 2010 but remains underused, with only five studies identified to date (3.5%; Fig. 2a). This telemetric method employs small, passive tags containing a unique identifier stored on a semiconductor chip. Tags are powered by a nearby reader and do not require batteries, making them extremely lightweight and suitable for attachment to very small insects [7, 8]. A fix is recorded each time a tracked insect passes within close proximity of a reader. Because detection typically requires a distance of less than 0.5 m, the reader often needs to be moved directly over the tag [29, 73]. RFID is most effective in laboratory or semi-natural setups, where readers can be strategically placed (e.g., [74]), for example, along tunnels [75], or burrow entrances [76]. For field tracking of free-ranging insects, detection can become labor-intensive, especially for very active individuals. Although unique identifiers theoretically allow researchers to reconstruct fine-scale trajectories and monitor multiple individuals simultaneously, in practice, continuous tracking is limited by high search intensity, leading to a decreasing number of recorded positions over time and space [29]. RFID is therefore suitable for capturing descriptive movement metrics but has only limited use for mechanistic goals such as habitat use or dispersal [73, 76]. Its applicability is mainly constrained by its short detection range and labor intensity. Altogether, RFID is suitable for species with limited dispersal and for microhabitat use in constrained areas, such as tunnels, burrow entrances, or enclosures. Its short detection range limits applicability for long-term, large-scale studies or movement predictions.

#### Harmonic radar

Harmonic radar is the oldest telemetric method in ground-dwelling insect research. First implemented in 1986 [77], it became widely used through the 1990s and early 2000s before radio telemetry became dominant, with a total of 16 records (11.3%) identified to date (Fig. 2a). This technology uses an active radar unit that functions as both transmitter and receiver. Insects are fitted with passive tags consisting of a small diode and a wire that retransmits the radar signal at half its original wavelength. These battery-free tags weigh only a few milligrams, making them suitable for small-bodied insects. However, they lack unique identifiers, which prevents the distinction of individuals when multiple tagged insects are tracked at the same time [78]. Manual visual confirmation is often necessary to resolve overlapping signals [44]. Detection range typically extends up to roughly 10 m, depending on antenna length [78], but tracking can continue for several months [30]. When movements are frequent, harmonic radar provides not only basic movement characteristics (e.g., [78–81]), but also long-term high-resolution movement data that allow for the reconstruction of detailed trajectories and the inference of behavioral states, habitat selection, and dispersal (e.g., [44, 58, 82]), with a potential for predictive movement studies. However, signal detection is strongly affected by moisture, dense vegetation, and uneven terrain, which makes field searches labor-intensive [78]. The method is also limited by the relatively short detection range, the absence of unique identifiers, and the high cost of the active radar unit [7]. Although harmonic radar is most commonly used to study migratory behaviour in butterflies (e.g., [83, 84]), in ground-dwelling insects, it is particularly well suited for tracking fine-scale trajectories, behavioral patterns, and habitat use over extended periods, enabling movement predictions. However, its application is limited in dense vegetation or wet conditions, and it does not allow reliable individual identification, which results in small sample sizes.

#### Radio telemetry

Very high frequency (VHF) radio telemetry currently represents the dominant telemetric method in ground-dwelling insect movement research, accounting for 17 records (12.1%; Fig. 2a). It uses active, battery-powered transmitters attached to insects that emit signals at unique frequencies, which are detected with a handheld receiver [7]. Originally developed for vertebrate studies, it has been effectively adapted for large-bodied, ground-dwelling insect species (e.g., [33, 85, 86]; Fig. 2b). The basic principle is to follow the transmitter signal until the tracked individual is located (with or without seeing it). Tracking efficiency depends on antenna length, terrain structure, vegetation density, and weather conditions. Short transmitter antennas reduce signal detectability and increase search time, while heavy rain, dense understory, and complex terrain may impair signal quality [87]. Experimental designs typically involve frequent relocation of multiple individuals over a maximum of two or three weeks, determined by transmitter battery life. Radio telemetry provides high spatial accuracy and enables detailed reconstruction of trajectories of multiple individuals at the same time [31], behavioral states [39], and habitat use [42], and may also support predictive modeling. Its critical limitations are transmitter weight and battery capacity. Some tags can exceed 50% of an insect’s body mass, restricting use to large species. Even the lightest available transmitters, which weigh below 0.2 g, typically last only seven days and can be used only once [7, 86]. Searching can be labor-intensive, and equipment costs remain relatively high compared to CMR or direct observations [88, 89]. To conclude, radio telemetry is well-suited for recording detailed movement paths, behavioral patterns, and habitat use of large species over the short to medium term. Moreover, it is the most advanced method in terms of technological advancement and development, including various cloud and software support. Its use is mostly limited by transmitter weight and battery life.

### Analytical approaches

The increasing accessibility of effective statistical programming environments such as R has accelerated the development of analytical frameworks for movement ecology [90]. Although many tools were originally designed for vertebrate GPS data, which are often noisy, non-linear, and strongly autocorrelated in space and time [91], they can be adapted for insects. A major challenge in the movement ecology of ground-dwelling insects is to clarify the motivation for the study. This may be especially true for telemetry studies, where attaching sophisticated equipment to insects does not automatically guarantee biologically meaningful results [86]. In our systematic search, almost half of all records (49.6%) reported only basic movement characteristics across five decades of research and the different tracking methods used (Fig. 2c and d). Such descriptive outputs are valuable, especially for rare, poorly known, or charismatic species, but ideally should be accompanied by more advanced analytical approaches [10, 91]. Small sample sizes, low recapture rates, and large inter-individual variation in movement further complicate analytical work. Methods suitable for ground-dwelling insects must therefore cope with high variability while remaining computationally efficient [91]. In addition, the temporal and spatial resolution must be fine enough to detect ecological processes, yet broad enough to capture full behavioral sequences.

Here, we organize analytical approaches along a descriptive–mechanistic–predictive continuum, reflecting increasing ecological inference and methodological complexity. Descriptive approaches quantify basic movement metrics such as daily dispersal, total path length, or net displacement. Mechanistic approaches relate movement to internal states (e.g., foraging, migration) or external drivers (e.g., habitat structure, microclimate). Predictive approaches build on mechanistic understanding to forecast movement across space and time. While some of these approaches are not yet widely implemented in ground-dwelling insect research, all are applicable to fine-scale movement data. To support implementation, we provide an overview of recommended R packages associated with each approach (Table 2). Although we do not include code examples, most packages provide vignettes and tutorials that can guide users through practical application.

**Table 2:** Overview of analytical approaches and relevant and actively maintained R packages (listed alphabetically) on CRAN or GitHub (last access: 7 December 2025) with potential applications to the movement ecology of ground-dwelling insects, particularly their fine-scale movement patterns. The framework is organized along a continuum from descriptive to mechanistic and predictive objectives, reflecting increasing analytical complexity and ecological insight

Research goal	Focus	Approach	*R* package	Reference
Descriptive	Quantifying basic movement characteristics	Data processing, simple evaluation, visualization	adehabitatLT	Calenge, [92]
			amt	Signer et al., [93]
			geosphere	Hijmans, [94]
			move2	McLean and Skowron Volponi, [95]
			trajr	Kranstauber et al., [96]
Mechanistic	Understanding processes behind observed movement patterns: home range, dispersal, behavior, habitat use	Home range	adehabitatHR	Calenge, [92]
			ctmm	Calabrese et al., [97]
			secr	Efford, [98]
		Dispersal kernels	dispfit	Proença-Ferreira et al., [99]
		Correlated random walk	aniMotum	Jonsen et al., [100]
			momentuHMM	McClintock & Michelot [101]
		Hidden Markov models	momentuHMM	McClintock & Michelot [101]
			moveHMM	Michelot et al., 2016
		Continuous-time models	aniMotum	Jonsen et al., [100]
			crawl	Johnson and London, [102]
			ctmm	Calabrese et al., [97]
		Resource and habitat selection	adehabitatHS	Calenge, [92]
			amt	Signer et al., [93]
			ResourceSelection	Lele et al., [103]
Predictive	Modeling future patterns: habitat connectivity, space use under environmental change	Long-term dispersal	crawl	Johnson and London, [102]
			steps	Visintin et al., [104]
		Landscape connectivity	gdistance	Etten, [105]
			grainscape	Chubaty et al., [106]
			resistanceGA	Peterman, [107]
		Climate change	crawl	Johnson and London, [102]
			steps	Visintin et al., [104]

#### Descriptive metrics and visualization

Summarizing basic movement characteristics is the first analytical step in movement studies. Descriptive metrics are simple quantitative values derived from fixes that characterize individual paths, including step length, turning angle, daily dispersal, total path length, and net displacement. They offer a rapid overview of movement behaviour and can be calculated even when only a few relocations are available. Such metrics are widely used to compare movement between sexes, developmental stages, species, or habitat types (e.g., [23, 61, 82]). Visual tools, such as trajectory maps, step-length histograms, or rose diagrams, complement these metrics by highlighting individual variability, directional trends, and site fidelity.

Despite their value, descriptive metrics are often treated as endpoints (Fig. 2c and d), and reports remain inconsistent. For instance, considering articles from the last decade (from 2016 to 2025; *n* = 37), only 45.9% reported values suitable for future meta-analysis and reproducibility, including mean daily dispersal with standard deviation or standard error and sample size. 18.9% of papers provided only partial data, while 35.2% did not provide any metrics that could be reused, instead reporting, for example, only maximum distances covered. Interestingly, studies with mechanistic goals often omit simple summary metrics, even though they can be easily added and interpreted. Reporting the number of relocations per individual, the sampling interval, and the tracking duration further improves comparability. Making raw or minimally processed movement data, including fixes’ coordinates, available also greatly increases its value for possible meta-analysis. Useful R packages for calculating and visualizing descriptive movement metrics include “adehabitatLT” [92], “amt” [93], “geosphere” [94], “trajr” [95], and “move2” [96].

#### Mechanistic movement models

Mechanistic research goals seek to explain the underlying processes that generate movement patterns resulting from behavioral decisions and environmental context, such as home range, dispersal, or habitat use (Table 2). In our systematic search, 47.6% of records focused on at least one mechanistic goal (Fig. 2c and d). Home range estimation is relatively uncommon in studies of ground-dwelling insects, yet feasible (e.g., [41, 65, 108]). Although the association with movement is indirect, home range reflects the cumulative rate of space use and shows exploration or habitat fidelity that directly results from movement. Minimum Convex Polygon (MCP) and Kernel Density Estimation (KDE) remain standard tools. MCP delineates the outer boundary of all locations but is sensitive to outliers and often overestimates space use [109, 110]. KDE instead estimates the probability distribution of space use and identifies core areas, yet requires larger and spatially rich datasets. Both approaches can be implemented in the “adehabitatHR” package [92], while the “ctmm” package offers more advanced KDEs that explicitly account for temporal autocorrelation [97]. Spatially Explicit Capture–Recapture (SECR) represents an alternative when movement paths are sparse or unavailable, but capture locations are known, and so movement probability in space and time, for example, from CMR pitfall trapping. The package “secr” allows flexible SECR model fitting and performs well in low-density populations with heterogeneous detectability [98, 111]. Dispersal kernels extend home range concepts by estimating the probability distribution of movement distances and capturing long-distance events [63, 64, 112]. They are widely used in habitat connectivity and conservation applications, such as defining sustainable habitat patches [63], and can be implemented via the “dispfit” package [99].

However, home range and dispersal kernels summarize space use in a relatively static way. To understand how movement changes over time, we need trajectory-based models that work directly with successive movement steps. This is particularly relevant for telemetry and observational studies where fixes are acquired at regular intervals. Early analytical efforts focused on trajectory geometry, starting with simple visual inspection (e.g., [32, 58]). These studies showed two distinct movement patterns in ground-dwelling insects: a *random walk*, characterized by short steps and frequent turns, which serves as a proxy for foraging, and *directed movement*, characterized by long steps and small turning angles, which can be interpreted as dispersal or migration away from unfavorable sites [32]. Other options are fractal dimensions, scale-independent metrics of path complexity, ranging from straight-line motion to infinitely long random walks [35, 40, 113], but with limited applicability in heterogeneous landscapes [114]. Correlated random walks offer a more robust alternative, assuming persistent directionality at fine scales and diffusive behaviour over longer scales [34, 37]. Lévy walks, characterized by short, clustered steps interspersed with occasional long displacements, have been proposed as efficient scale-independent strategies for sparse and patchy resources [115, 116]. However, similar patterns can arise from multiple underlying processes, including habitat structure or sampling design. Lévy models, therefore, risk oversimplifying behaviour [117, 118]. These limitations highlight the need for mechanistic, behaviour-informed approaches. State-switching models, such as hidden Markov models (HMMs), address this problem by segmenting trajectories into behavioral states (e.g., random walk, directed movement) based on step lengths and turn angles set before the analysis [38, 39, 119]. HMMs estimate switching probabilities between behavioral states, but do not inherently model habitat-driven decisions [120]. Habitat use can be assessed post hoc, for example, by comparing the frequency of behavioral states across habitat types using generalized linear mixed models [86]. HMMs can be implemented via the “moveHMM” package [38] or its extended version, “momentuHMM” [101], which also supports biased and correlated random walks.

When relocation intervals are irregular or uneven, due to technical or logistical constraints, continuous-time models are more suitable. These models treat movement as a stochastic process, accommodate irregular sampling, and naturally incorporate autocorrelation in speed and direction [121–123]. They are typically based on diffusion processes, such as Brownian [124] and Ornstein-Uhlenbeck [125]. To date, continuous-time models have not been applied to ground-dwelling insects due to demanding data requirements, although their flexibility makes them promising for future studies [91]. The packages that can handle the continuous-time movement models are “aniMotum” [100], “crawl” [102], and “ctmm” [97].

Movement can also be strongly shaped by habitat structure. A common but simple approach is to use step lengths as a continuous response in generalized linear mixed models to test the effect of different habitat types (e.g., [31, 85, 126, 127]). While useful for habitat use questions, this approach does not account for autocorrelation between consecutive fixes and therefore cannot be interpreted as trajectory-level movement behaviour. More formal frameworks for analyzing habitat use include compositional analysis, which compares the actual use of different habitat types to their overall availability [42, 128]. Another widely used approach is resource-selection functions, which compare habitat-related covariates at fixes where the individual was observed with covariates at random fixes within the available area [129, 130]. However, both approaches assume independence among successive fixes (i.e., relocations are spaced sufficiently apart in time to allow an individual to potentially reach any location in the landscape), an assumption rarely met in fine-scale ground-dwelling insect movement [40, 131]. Step-selection functions overcome this limitation by comparing environmental covariates at observed fixes to randomly generated alternative fixes while retaining step length and turning angle distributions, thus accounting for autocorrelation [93, 132]. These analyses can be implemented using several R packages, such as “adehabitatHS” for compositional analysis [92], “resourceSelection” for resource-selection functions [103], and “amt” for step-selection functions.

#### Predictive and applied movement ecology

Predictive models aim to forecast movement across new spatial or temporal scenarios by integrating individual traits (e.g., behavior, physiology) with environmental variables [12, 133, 134]. While these models are increasingly applied to flying insects (e.g., 135, 136, 137], they remain rare for ground-dwelling insects due to sampling constraints and poor data robustness, with only four publications (2.8% of all records) based on the CMR method to date (Fig. 2c and d). Main limitations of the application of predictive approaches include incomplete lifetime tracking [12], seasonal and life-history variation in movement patterns [114], and species-specific responses to microhabitats and local barriers, which hinder broad generalization.

Still, predictive approaches are urgently needed in the context of climate change, habitat fragmentation, and other disturbances, all of which are reshaping species distributions [13]. With robust datasets, R packages originally developed for vertebrates, such as “crawl” and “steps” [104], could possibly model long-term trajectories of ground-dwelling insects across heterogeneous landscapes. For studies of landscape connectivity, packages such as “gdistance” [105], “resistanceGA” [107], and “grainscape” [106] can estimate resistance surfaces, functional connectivity, and movement costs. This is especially relevant for ground-dwelling insects moving through structurally complex ground layers or responding to small-scale barriers, such as roads. Under climate change scenarios, “crawl” and “steps” packages can simulate whether species can adapt to shifting microclimates or recolonize suitable habitat patches. These simulations can also be integrated into GIS platforms, such as GRASS GIS [138], linking movement predictions with spatial environmental layers to inform species distribution modeling and conservation planning under future scenarios.

## Future prospects

One of the central goals of movement ecology is to understand and predict how individuals will respond to changes in their environment [13]. For ground-dwelling insects, this requires moving beyond basic descriptive metrics toward a more mechanistic and predictive understanding of behavior under habitat alteration, disturbances, and climate change. Achieving this goal depends on robust movement data collected over sufficiently long periods and large spatial scales, yet detailed enough to interpret individual behavior and habitat use [139].

A key factor enabling such robust datasets is the development of smaller, more affordable tracking devices capable of monitoring individuals over their entire adult lifespan. Ideally, these tools should allow remote data collection to reduce labor-intensive fieldwork. While some remote tracking techniques (e.g., [9, 20, 21, 140]), including mobile aerial units [49], have been applied to flying insects, the cryptic lifestyle and fine-scale movements of ground-dwelling species call for more adjusted methods.

Once movement data are collected, minimum reporting standards and open data sharing are essential to enable cross-study consistency and synthesis. At a minimum, studies should report daily dispersal (distance covered in one day), together with measures of variation (SD or SE) and sample size. Supplementary movement information, such as step lengths, turning angles, coordinates of relocations, number of relocations per individual, sampling interval, and tracking duration, can further improve comparability and also facilitate meta-analyses. Making raw or minimally processed movement data publicly available, for example, through repositories such as Movebank (https://www.movebank.org), allows integration across species, sites, and experimental designs. Nevertheless, Movebank hosts currently only a single published (not-ground-dwelling) insect dataset, tracking the nocturnally migrating moth (*Acherontia atropos*), highlighting the under-representation of insects in global movement databases [141].

Finally, movement data must be integrated with the environmental context. Movement behavior is influenced by a range of biotic and abiotic factors, from vegetation structure and temperature to resource availability and species interactions. Without these contexts, movement data remain largely descriptive. Analytical workflows that link movement trajectories to landscape and local habitat features, particularly under dynamic conditions, are essential for developing ecologically meaningful predictions and for advancing predictive movement ecology.

## Conclusions

In light of the current biodiversity crisis, there is increasing pressure to apply refined, non-lethal, and ecologically meaningful methods in entomological research [4]. In this review, we summarized the sampling methods and analytical approaches available for studying the movement ecology of ground-dwelling insects, outlining their strengths, limitations, and suitability for different research goals. Because movement behaviour and spatial scale vary widely among taxa, no single method fits all situations. Method choice must therefore be driven by research goal, and combining approaches often yields the most informative results. Individual-based movement studies offer valuable insights into the biology and ecology of particular species and have strong potential to complement, rather than replace, community-level approaches. They are especially useful for behavioral ecology and can reveal mechanisms behind patterns detected at the assemblage level. Integrating both perspectives may provide a more complete understanding of ground-dwelling insect responses to environmental change. Future progress will depend on improving the spatial and temporal resolution of movement data, advancing miniaturized tracking technologies, and better linking movement trajectories with environmental context. Equally important is the adoption of minimum reporting standards and greater data openness, which will facilitate comparison and synthesis across studies. With these steps, ground-dwelling insects can play a more prominent role in predictive and applied movement ecology.

## Supplementary Information

Below is the link to the electronic supplementary material.

Supplementary Material 1

## Data Availability

All data used are already published in the relevant publications provided in the reference list at the end of the manuscript and in the Supplementary Material S2.

## Abbreviations

CMR: Capture-Mark-Recapture
GPS: Global Positioning System
HMM: Hidden Markov Model
KDE: Kernel Density Estimation
MCP: Minimum Convex Polygon
SECR: Spatially Explicit Capture–Recapture
RFID: Radio-Frequency Identification
UTM: Universal Transverse Mercator
VHF: Very High Frequency
