# Supplementary material for: Challenges and approaches in tracking and analyzing movement of ground-dwelling insects
Source: Mov Ecol. 2026 Mar 4;14:25. doi: 10.1186/s40462-026-00637-x (PMC13067757; doi:10.1186/s40462-026-00637-x)
Supplement: Supplementary file 1 — Supplementary Material 1 [file 40462_2026_637_MOESM1_ESM.docx]

**Online supplementary material**

**Jana Růžičková and Zoltán Elek:** Challenges and approaches in tracking and analyzing of ground-dwelling insects

**Supplementary Material S1. Literature search and selection**

This section provides the full search string used to identify studies on the movement of ground-dwelling insects.The search string targeted three concept groups: movement-related terms, terrestrial insect taxa, and methods used to quantify individual movement under field conditions.

**Web of Science search parameters**

Timespan: 1975-2025

Collection: Web of Science Core collection

Date of search: 27-10-2025

Article type: Article, Proceeding Papers, Review and Book Chapters

Categories: Ecology, Entomology, Zoology, Biodiversity Conservation

Date of search: 27-10-2025

**Search string:**

Topic = (

("movement*" OR "trajectory" OR "dispersal" OR "activity pattern*" OR "habitat use" OR "home range" OR "spatial behaviour" OR "spatial behavior" OR "movement path")

AND

("insect*" OR "beetle*" OR "coleopter*" OR "carabid*" OR "staphylinid*" OR "tenebrionid*" OR "orthopter*" OR "phasmatodea*" OR "ground-dwelling" OR "terrestrial arthropod*" OR "flightless insect*")

AND

("mark-recapture" OR "mark recapture" OR "telemetry" OR "radio-tracking" OR "radio telemetry" OR "RFID" OR "harmonic radar" OR "fluorescent powder" OR "tracking*" OR "direct observation" OR "pitfall trap*" OR "quantifying")

)

**Supplementary Material S2: Literature included in the systematic review in alphabetic order**

1. Allema, A. B., Van Der Werf, W., Groot, J. C. J., Hemerik, L., Gort, G., Rossing, W. A. H., & van Lenteren, J. C. (2015). Quantification of motility of carabid beetles in farmland. Bulletin of Entomological Research, 105(2), 234-244. <https://doi.org/10.1017/S0007485315000012>
2. Allema, B., van der Werf, W., van Lenteren, J. C., Hemerik, L., & Rossing, W. A. (2014). Movement behaviour of the carabid beetle Pterostichus melanarius in crops and at a habitat interface explains patterns of population redistribution in the field. PloS one, 9(12), e115751. <https://doi.org/10.1371/journal.pone.0115751>
3. Anderson, M. E., Harman, R. R., & Kim, T. N. (2024). Ground beetle movement is deterred by habitat edges: a mark-release-recapture study on the effectiveness of border crops in an agricultural landscape. Journal of Insect Science, 24(3), 24. <https://doi.org/10.1093/jisesa/ieae062>
4. Anselmo, L. (2022). A field study on Saga pedo (Ensifera, Tettigoniidae, Saginae). Journal of Orthoptera Research, 31(1), 41-46. <https://doi.org/10.3897/jor.31.69425>
5. Baars, M. A. (1979). Catches in pitfall traps in relation to mean densities of carabid beetles. Oecologia, 41(1), 25-46. <https://doi.org/10.1007/BF00344835>
6. Baars, M. A. (1979). Patterns of movement of radioactive carabid beetles. Oecologia, 44(1), 125-140. <https://doi.org/10.1007/BF00346411>
7. Balogh, S. L., Björklund, N., Huber, D. P., & Lindgren, B. S. (2020). Random and directed movement by Warren root collar weevils (Coleoptera: Curculionidae), relative to size and distance of host lodgepole pine trees. Journal of Insect Science, 20(4), 9. <https://doi.org/10.1093/jisesa/ieaa063>
8. Bangert, R. K., & Slobodchikoff, C. N. (2004). Prairie dog engineering indirectly affects beetle movement behavior. Journal of Arid Environments, 56(1), 83-94. <https://doi.org/10.1016/S0140-1963(02)00322-1>
9. Bérces, S., & Elek, Z. (2013). Overlapping generations can balance the fluctuations in the activity patterns of an endangered ground beetle species: long-term monitoring of Carabus hungaricus in Hungary. Insect Conservation and Diversity, 6(3), 290-299. <https://doi.org/10.1111/j.1752-4598.2012.00218.x>
10. Bérces, S., & Růžičková, J. (2019). Habitat use of an endangered beetle Carabus hungaricus assessed via radio telemetry. Acta Zoologica Academiae Scientiarum Hungaricae, 65(4), 335-348. <https://doi.org/10.17109/AZH.65.4.335.2019>
11. Bérces, S., Fülöp, D., & Samu, F. (2022). Life history adaptations of Carabus populations in a suburban park: A capture-recapture case study. Global Ecology and Conservation, 35, e02086. <https://doi.org/10.1016/j.gecco.2022.e02086>
12. Berggren, Å. (2005). The effect of conspecifics on individual male movement in Roesel's bush cricket, Metrioptera roeseli. Ecological Entomology, 30(4), 480-483. <https://doi.org/10.1111/j.0307-6946.2005.00709.x>
13. Berggren, Å. (2004). Impact of grazing on individual male movement in Roesel's bush-cricket Metrioptera roeseli: One possible clue to species range expansion. Journal of Insect Behavior, 17(4), 419-429. <https://doi.org/10.1023/B:JOIR.0000042531.27859.ac>
14. Bertoncelj, I., & Dolman, P. M. (2013). The matrix affects trackway corridor suitability for an arenicolous specialist beetle. Journal of insect conservation, 17(3), 503-510. <https://doi.org/10.1007/s10841-012-9533-9>
15. Best, R. L., Beegle, C. C., Owens, J. C., & Ortiz, M. (1981). Population density, dispersion, and dispersal estimates for Scarites substriatus, Pterostichus chalcites, and Harpalus pennsylvanicus (Carabidae) in an Iowa cornfield. Environmental Entomology, 10(6), 847-856. <https://doi.org/10.1093/ee/10.6.847>
16. Blight, O., Geslin, B., Mottet, L., & Albert, C. H. (2023). Potential of RFID telemetry for monitoring ground-dwelling beetle movements: A Mediterranean dry grassland study. Frontiers in Ecology and Evolution, 11, 1040931. <https://doi.org/10.3389/fevo.2023.1040931>
17. Boisseau, R. P., Ero, M. M., Makai, S., Bonneau, L. J., & Emlen, D. J. (2020). Sexual dimorphism divergence between sister species is associated with a switch in habitat use and mating system in thorny devil stick insects. Behavioural Processes, 181, 104263. <https://doi.org/10.1016/j.beproc.2020.104263>
18. Brazee, R. D., Miller, E. S., Reding, M. E., Klein, M. G., Nudd, B., & Zhu, H. (2005). A transponder for harmonic radar tracking of the black vine weevil in behavioral research. Transactions of the ASAE, 48(2), 831-838. <https://doi.org/10.13031/2013.18306>
19. Brouwers, N. C., & Newton, A. C. (2010). Movement analyses of wood cricket (Nemobius sylvestris)(Orthoptera: Gryllidae). Bulletin of entomological research, 100(6), 623-634. <https://doi.org/10.1017/S0007485309990332>
20. Cerda, Y., Grez, A. A., & Simonetti, J. A. (2015). The role of the understory on the abundance, movement and survival of Ceroglossus chilensis in pine plantations: an experimental test. Journal of Insect Conservation, 19(1), 119-127. <https://doi.org/10.1007/s10841-015-9752-y>
21. Chappell, E. M., Webb, D. S., & Tonkin, J. D. (2014). Notes on sexual size dimorphism, sex ratio and movements of adult ground weta Hemiandrus maculifrons (Walker)(Orthoptera: Anostostomatidae). New Zealand Entomologist, 37(2), 83-92. <https://doi.org/10.1080/00779962.2013.856377>
22. Charrier, S., Petit, S., & Burel, F. (1997). Movements of Abax parallelepipedus (Coleoptera, Carabidae) in woody habitats of a hedgerow network landscape: a radio-tracing study. Agriculture, ecosystems & environment, 61(2-3), 133-144. <https://doi.org/10.1016/S0167-8809(96)01101-2>
23. Crist, T. O., & Wiens, J. A. (1995). Individual movements and estimation of population size in darkling beetles (Coleoptera: Tenebrionidae). Journal of animal ecology, 64(6), 733-746. <https://doi.org/10.2307/5852>
24. Crist, T. O., Guertin, D. S., Wiens, J. A., & Milne, B. T. (1992). Animal movement in heterogeneous landscapes: an experiment with Eleodes beetles in shortgrass prairie. Functional Ecology, 6(5), 536-544. <https://doi.org/10.2307/2390050>
25. De Gasperis, S. D., Passacantilli, C., Zan, L. D., & Carpaneto, G. M. (2016). Overwintering ability and habitat preference of Morimus asper: a two-year mark-recapture study with implications for conservation and forest management. Journal of Insect Conservation, 20(5), 821-835. <https://doi.org/10.1007/s10841-016-9913-7>
26. Diekötter, T., Speelmans, M., Dusoulier, F., Van Wingerden, W. K., Malfait, J. P., Crist, T. O., Edwards, P. J., & Dietz, H. (2007). Effects of landscape structure on movement patterns of the flightless bush cricket Pholidoptera griseoaptera. Environmental Entomology, 36(1), 90-98. <https://doi.org/10.1603/0046-225X(2007)36[90:EOLSOM]2.0.CO;2>
27. Diekötter, T., Csencsics, D., Rothenbühler, C., Billeter, R., & Edwards, P. J. (2005). Movement and dispersal patterns in the bush cricket Pholidoptera griseoaptera: the role of developmental stage and sex. Ecological Entomology, 30(4), 419-427. <https://doi.org/10.1111/j.0307-6946.2005.00714.x>
28. Drees, C., Matern, A., & Assmann, T. (2008). Behavioural patterns of nocturnal carabid beetles determined by direct observations under red-light conditions. In: Back to the Roots and Back to the Future (pp. 421-435). Pensoft Publishers. Sofia–Moscow.
29. Eggers, B., Matern, A., Drees, C., Eggers, J., Haerdtle, W., & Assmann, T. (2010). Value of semi‐open corridors for simultaneously connecting open and wooded habitats: A case study with ground beetles. Conservation Biology, 24(1), 256-266. <https://doi.org/10.1111/j.1523-1739.2009.01295.x>
30. Elek, Z., Růžičková, J., & Ódor, P. (2021). Individual decisions drive the changes in movement patterns of ground beetles between forestry management types. Biologia, 76(11), 3287-3296. <https://doi.org/10.1007/s11756-021-00805-x>
31. Elek, Z., Drag, L., Pokluda, P., Čížek, L., & Bérces, S. (2014). Dispersal of individuals of the flightless grassland ground beetle, Carabus hungaricus (Coleoptera: Carabidae), in three populations and what they tell us about mobility estimates based on mark-recapture. European Journal of Entomology, 111(5), 663-668. <https://doi.org/10.14411/eje.2014.080>
32. Eufrázio, S., Oliveira, A., Miralto, O., Medinas, D., Silva, C., Sá, C., Mira, A., & Salgueiro, P. A. (2020). Unraveling the dynamics of a ground‐dwelling beetle population exposed to quarry exploitation and restoration practices. Restoration Ecology, 28(3), 697-705. <https://doi.org/10.1111/rec.13056>
33. Fountain, E. D., Wiseman, B. H., Cruickshank, R. H., & Paterson, A. M. (2013). The ecology and conservation of Hadramphus tuberculatus (pascoe 1877)(Coleoptera: Curculionidae: Molytinae). Journal of Insect Conservation, 17(4), 737-745. <https://doi.org/10.1007/s10841-013-9557-9>
34. Fournier, E., & Loreau, M. (2001). Activity and satiation state in Pterostichus melanarius: an experiment in different agricultural habitats. Ecological entomology, 26(3), 235-244. <https://doi.org/10.1046/j.1365-2311.2001.00314.x>
35. Frampton, G. K., Çilgi, T., Fry, G. L., & Wratten, S. D. (1995). Effects of grassy banks on the dispersal of some carabid beetles (Coleoptera: Carabidae) on farmland. Biological conservation, 71(3), 347-355. <https://doi.org/10.1016/0006-3207(94)00072-X>
36. Gerber, A. S., & Templeton, A. R. (1996). Population sizes and within-deme movement of Trimerotropis saxatilis (Acrididae), a grasshopper with a fragmented distribution. Oecologia, 105(3), 343-350. <https://doi.org/10.1007/BF00328737>
37. Gordon, P. L., & McKinlay, R. G. (1986). Dispersal of ground beetles in a potato crop; a mark-release study. Entomologia Experimentalis et Applicata, 40: 104-105. <https://doi.org/10.1111/j.1570-7458.1986.tb02163.x>
38. Gwynne, D. T., & Kelly, C. D. (2018). Successful use of radiotransmitters in tracking male tree wētā Hemideina crassidens (Orthoptera: Tettigonioidea: Anostostomatidae). New Zealand Entomologist, 41(1), 25-28. <https://doi.org/10.1080/00779962.2018.1501138>
39. Hatten, T. D., Bosque‐Pérez, N. A., Johnson‐Maynard, J., & Eigenbrode, S. D. (2007). Tillage differentially affects the capture rate of pitfall traps for three species of carabid beetles. Entomologia Experimentalis et Applicata, 124(2), 177-187. <https://doi.org/10.1111/j.1570-7458.2007.00566.x>
40. Haynes, K. J., Diekötter, T., & Crist, T. O. (2007). Resource complementation and the response of an insect herbivore to habitat area and fragmentation. Oecologia, 153(3), 511-520. <https://doi.org/10.1007/s00442-007-0749-4>
41. Heidinger, I. M. M., Poethke, H. J., Bonte, D., & Hein, S. (2009). The effect of translocation on movement behaviour—a test of the assumptions of behavioural studies. Behavioural Processes, 82(1), 12-17. <https://doi.org/10.1016/j.beproc.2009.03.001>
42. Holland, J. M., Begbie, M., Birkett, T., Southway, S., Thomas, S. R., Alexander, C. J., & Thomas, C. F. G. (2004). The spatial dynamics and movement of Pterostichus melanarius and P. madidus (Carabidae) between and within arable fields in the UK. International Journal of Ecology and Environmental Sciences, 30, 35-53.
43. Holuša, J., Kočárek, P., & Vlk, R. (2013). Monitoring and conservation of Saga pedo (Orthoptera: Tettigoniidae) in an isolated nothwestern population. Journal of insect conservation, 17(4), 663-669. <https://doi.org/10.1007/s10841-013-9550-3>
44. Jamieson, I. G., Forbes, M. R., & McKnight, E. B. (2000). Mark-recapture study of mountain stone weta Hemideina maori (Orthoptera: Anostostomatidae) on rock tor'islands'. New Zealand Journal of Ecology, 24(2), 209-214.
45. Jensen, T. S., Dyring, L., Kristensen, B., Nielsen, B. O., & Rasmussen, E. R. (1989). Spring dispersal and summer habitat distribution of Agonum dorsale (Coleoptera, Carabidae). Pedobiologia, 33(3), 155-165.
46. Joern, A. (1983). Small-scale displacements of grasshoppers (Orthoptera: Acrididae) within arid grasslands. Journal of the Kansas Entomological Society,56(2), 131-139.
47. Johnson, A. R., Milne, B. T., & Wiens, J. A. (1992). Diffusion in Fractcal landscapes: simulations and experimental studies of tenebrionid beetle movements. Ecology, 73(6), 1968-1983. <https://doi.org/10.2307/1941448>
48. Joyce, K. A., Holland, J. M., & Doncaster, C. P. (1999). Influences of hedgerow intersections and gaps on the movement of carabid beetles. Bulletin of Entomological Research, 89(6), 523-531. <https://doi.org/10.1017/S000748539900067X>
49. Kagawa, Y., & Maeto, K. (2009). Spatial population structure of the predatory ground beetle Carabus yaconinus (Coleoptera: Carabidae) in the mixed farmland-woodland satoyama landscape of Japan. European Journal of Entomology, 106(3), 385. <https://doi.org/10.14411/eje.2009.049>
50. Kelly, C. D., & Gwynne, D. T. (2023). Effect of body condition on mobility and mating success in a wild population of the scramble polygynous Cook Strait giant weta. Behavioral Ecology and Sociobiology, 77(1), 5. <https://doi.org/10.1007/s00265-022-03278-3>
51. Kelly, C. D., Gagnon, R., Larrivée, M., & Saint-Germain, M. (2024). Demography, behavior, and morphology of the Northern Barrens tiger beetle, Cicindela patruela patruela (Coleoptera: Carabidae), on Île-aux-Allumettes, Quebec. Annals of the Entomological Society of America, 117(1), 36-43. <https://doi.org/10.1093/aesa/saad035>
52. Kennedy, P. J. (1994). The distribution and movement of ground beetles in relation to set-aside arable land. In Carabid Beetles: Ecology and Evolution (pp. 439-444). Springer. Dordrecht, Netherlands. <https://doi.org/10.1007/978-94-017-0968-2_66>
53. Kho, J. W., Kim, Y. J., Kim, H., Hong, S. H., Lee, Y. S., Park, J. S., & Lee, D. H. (2024). Development of underground detection system using a metal detector and aluminum tag for Copris ochus (Coleoptera: Scarabaeidae). Journal of Insect Science, 24(3), 27. <https://doi.org/10.1093/jisesa/ieae067>
54. Kindvall, O. (1999). Dispersal in a metapopulation of the bush cricket, Metrioptera bicolor (Orthoptera: Tettigoniidae). Journal of animal Ecology, 68(1), 172-185. <https://doi.org/10.1046/j.1365-2656.1999.00273.x>
55. Kiss, J., Németh, Z., Kosztolányi, A., & Barta, Z. (2020). Differential movement and activity patterns of sexes in a biparental beetle during the reproductive season. Ecological Entomology, 45(6), 1504-1508. <https://doi.org/10.1111/een.12920>
56. Klingenberg, M. D., Björklund, N., & Aukema, B. H. (2010). Seeing the forest through the trees: differential dispersal of Hylobius warreni within modified forest habitats. Environmental Entomology, 39(3), 898-906. <https://doi.org/10.1603/EN08269>
57. Koivula, M. J., & Vermeulen, H. J. (2005). Highways and forest fragmentation–effects on carabid beetles (Coleoptera, Carabidae). Landscape Ecology, 20(8), 911-926. <https://doi.org/10.1007/s10980-005-7301-x>
58. Kromp, B., & Nitzlader, M. (1995). Dispersal of ground beetles in a rye field in Vienna, Eastern Austria. Arthropod natural enemies in arable land, 1: Density, spatial heterogeneity and dispersal. 269-277.
59. Kujawa, K., Sobczyk, D., & Kajak, A. (2006). Dispersal of Harpalus rufipes (Degeer)(Carabidae) between shelterbelt and cereal field. Polish Journal of Ecology, 54(2), 243-252.
60. László, M., Laczi, M., & Szövényi, G. (2025). Conservation on the edge: unravelling critical drivers behind the decline of a mountain grasshopper’s marginal populations. Journal of Insect Conservation, 29(3), 44. <https://doi.org/10.1007/s10841-025-00682-8>
61. Loreau, M., & Nolf, C. L. (1993). Occupation of space by the carabid beetle Abax ater. Acta Oecologica, 14(2), 247-258.
62. Lorch, P. D., & Gwynne, D. T. (2000). Radio-telemetric evidence of migration in the gregarious but not the solitary morph of the Mormon cricket (Anabrus simplex: Orthoptera: Tettigoniidae). Naturwissenschaften, 87(8), 370-372. <https://doi.org/10.1007/s001140050743>
63. Lorch, P. D., Sword, G. A., Gwynne, D. T., & Anderson, G. L. (2005). Radiotelemetry reveals differences in individual movement patterns between outbreak and non‐outbreak Mormon cricket populations. Ecological Entomology, 30(5), 548-555. <https://doi.org/10.1111/j.0307-6946.2005.00725.x>
64. Lövei, G. L., Stringer, I. A., Devine, C. D., & Cartellieri, M. (1997). Harmonic radar-a method using inexpensive tags to study invertebrate movement on land. New Zealand Journal of Ecology, 187-193.
65. Lys, J. A., & Nentwig, W. (1992). Augmentation of beneficial arthropods by strip-management: 4. Surface activity, movements and activity density of abundant carabid beetles in a cereal field. Oecologia, 92(3), 373-382. <https://doi.org/10.1007/BF00317463>
66. Lys, J. A., & Nentwig, W. (1991). Surface activity of carabid beetles inhabiting cereal fields. Seasonal phenology and the influence of farming operations on five abundant species. Pedobiologia, 35(3), 129-138.
67. Madeira, F., & Pons, X. (2016). Rubidium marking reveals different patterns of movement in four ground beetle species (Col., Carabidae) between adjacent alfalfa and maize. Agricultural and Forest Entomology, 18(2), 99-107. <https://doi.org/10.1111/afe.12141>
68. Maes, D., Ghesquiere, A., Logie, M., & Bonte, D. (2006). Habitat use and mobility of two threatened coastal dune insects: implications for conservation. Journal of insect conservation, 10(2), 105-115. <https://doi.org/10.1007/s10841-006-6287-2>
69. Machial, L. A., Lindgren, B. S., Steenweg, R. W., & Aukema, B. H. (2012). Dispersal of Warren root collar weevils (Coleoptera: Curculionidae) in three types of habitat. Environmental entomology, 41(3), 578-586. <https://doi.org/10.1603/EN11169>
70. Martay, B., Robertshaw, T., Doberski, J., & Thomas, A. (2014). Does dispersal limit beetle re‐colonization of restored fenland? A case study using direct measurements of dispersal and genetic analysis. Restoration Ecology, 22(5), 590-597. <https://doi.org/10.1111/rec.12118>
71. Mascanzoni, D., & Wallin, H. (1986). The harmonic radar: a new method of tracing insects in the field. Ecological Entomology, 11(4), 387-390. <https://doi.org/10.1111/j.1365-2311.1986.tb00317.x>
72. Mason, P. L., Nichols, R. A., & Hewitt, G. M. (1995). Philopatry in the alpine grasshopper, Podisma pedestris: a novel experimental and analytical method. Ecological entomology, 20(2), 137-145. <https://doi.org/10.1111/j.1365-2311.1995.tb00439.x>
73. Matenaar, D., Bröder, L., Bazelet, C. S., & Hochkirch, A. (2014). Persisting in a windy habitat: population ecology and behavioral adaptations of two endemic grasshopper species in the Cape region (South Africa). Journal of Insect Conservation, 18(3), 447-456. <https://doi.org/10.1007/s10841-014-9654-4>
74. Mauremooto, J. R., Wratten, S. D., Worner, S. P., & Fry, G. L. A. (1995). Permeability of hedgerows to predatory carabid beetles. Agriculture, ecosystems & environment, 52(2-3), 141-148. <https://doi.org/10.1016/0167-8809(94)00548-S>
75. McIntyre, N. E. (2000). Community structure of Eleodes beetles (Coleoptera: Tenebrionidae) in the shortgrass steppe: scale-dependent uses of heterogeneity. Western North American Naturalist, 60(1), 1-15.
76. Mo, J., & Stevens, M. M. (2013). Vertical and horizontal movements of Fuller's rose weevil (Coleoptera: Curculionidae) in Australian citrus groves. Entomological Science, 16(1), 26-33. <https://doi.org/10.1111/j.1479-8298.2012.00533.x>
77. Narisu, N., Lockwood, J. A., & Schell, S. P. (1999). A novel mark-recapture technique and its application to monitoring the direction and distance of local movements of rangeland grasshoppers (Orthoptera: Acrididae) in the context of pest management. Journal of Applied Ecology, 36(4), 604-617.
78. Negro, M., Caprio, E., Leo, K., Maritano, U., Roggero, A., Vacchiano, G., Palestrini, C. & Rolando, A. (2017). The effect of forest management on endangered insects assessed by radio-tracking: The case of the ground beetle Carabus olympiae in European beech Fagus sylvatica stands. Forest ecology and management, 406, 125-137. <https://doi.org/10.1016/j.foreco.2017.09.065>
79. Negro, M., Casale, A., Migliore, L. U. C. A., Palestrini, C., & Rolando, A. (2008). Habitat use and movement patterns in the endangered ground beetle species, Carabus olympiae (Coleoptera: Carabidae). European Journal of Entomology, 105(1), 105-112. <https://doi.org/10.14411/eje.2008.015>
80. Niehues, F. J., Hockmann, P., & Weber, F. (1996, January). Genetics and dynamics of a Carabus auronitens metapopulation in the Westphalian Lowlands (Coleoptera, Carabidae). Annales Zoologici Fennici, 33(1), 85-96.
81. Niemelä, P. T., Tiso, S., & Dingemanse, N. J. (2021). Density-dependent individual variation in male attractiveness in a wild field cricket. Behavioral Ecology, 32(4), 707-716. <https://doi.org/10.1093/beheco/arab009>
82. Nuhlíčková, S., Svetlík, J., Kaňuch, P., Krištín, A., & Jarčuška, B. (2024). Movement patterns of the endemic flightless bush-cricket, Isophya beybienkoi. Journal of Insect Conservation, 28(1), 141-150. <https://doi.org/10.1007/s10841-023-00529-0>
83. Nwana, I. E. (1984). The dispersal of the variegated grasshopper, Zonocerus variegatus (Linnaeus)(Orthoptera, Acridoidea, Pyrgomorphidae), in open fields and cultivated farms. International Journal of Tropical Insect Science, 5(4), 273-278. <https://doi.org/10.1017/S1742758400001569>
84. Oh, S. N., Choi, E. Y., Choi, J. B., Han, E. J., Woo, D. G., & Park, J. K. (2022). The role of the eco–corridor for the walking beetles in Chupungryeong, Korea. Journal of Asia-Pacific Biodiversity, 15(4), 598-602. <https://doi.org/10.1016/j.japb.2022.09.007>
85. O'Neal, M. E., Landis, D. A., Rothwell, E., Kempel, L., & Reinhard, D. (2004). Tracking insects with harmonic radar: a case study. American Entomologist, 50(4), 212-218. <https://doi.org/10.1093/ae/50.4.212>
86. Perry, K. I., Sivakoff, F. S., Wallin, K. F., Wenzel, J. W., & Herms, D. A. (2021). Forest disturbance and arthropods: small‐scale canopy and understory disturbances alter movement of mobile arthropods. Ecosphere, 12(11), e03771. <https://doi.org/10.1002/ecs2.3771>
87. Perry, K. I., Wallin, K. F., Wenzel, J. W., & Herms, D. A. (2017). Characterizing movement of ground-dwelling arthropods with a novel mark-capture method using fluorescent powder. Journal of Insect Behavior, 30(1), 32-47. <https://doi.org/10.1007/s10905-017-9598-0>
88. Petit, S. (1994). Diffusion of forest carabid beetles in hedgerow network landscapes. In Carabid beetles: ecology and evolution (pp. 337-341). Springer. Dordrecht, Netherlands. <https://doi.org/10.1007/978-94-017-0968-2_51>
89. Plewińska, B. (2007). The effect of food odour on food preference, activity and density of dung beetle Geotrupes stercorosus (Scriba, 1791) in a mixed coniferous forest. Polish Journal of Ecology, 55(3), 495-509.
90. Pope, T., Gundalai, E., Elliott, L., Blackshaw, R., Hough, G., Wood, A., Bennison, J., Prince, G., & Chandler, D. (2015). Recording the movement of adult vine weevil within strawberry crops using radio frequency identification tags. Journal of Berry Research, 5(4), 197-206. <https://doi.org/10.3233/JBR-150102>
91. Ranjha, M. H., & Irmler, U. (2014). Movement of carabids from grassy strips to crop land in organic agriculture. Journal of insect conservation, 18(3), 457-467. <https://doi.org/10.1007/s10841-014-9657-1>
92. Riecken, U., & Raths, U. (1996, January). Use of radio telemetry for studying dispersal and habitat use of Carabus coriaceus L. Annales Zoologici Fennici 33(1), 109-116.
93. Rijnsdorp, A. D. (1980). Pattern of movement in and dispersal from a Dutch forest of Carabus problematicus Hbst.(Coleoptera, Carabidae). Oecologia, 45(2), 274-281. <https://doi.org/10.1007/BF00346470>
94. Roslin, T. (2000). Dung beetle movements at two spatial scales. Oikos, 91(2), 323-335. <https://doi.org/10.1034/j.1600-0706.2000.910213.x>
95. Russek, L. A., Mansilla, C. L., Crespin, S. J., Simonetti, J. A., & Grez, A. A. (2017). Accompanying vegetation in young Pinus radiata plantations enhances recolonization by Ceroglossus chilensis (Coleoptera: Carabidae) after clearcutting. Journal of Insect Conservation, 21(5), 943-950. <https://doi.org/10.1007/s10841-017-0033-9>
96. Růžičková, J., & Elek, Z. (2021). Recording fine‐scale movement of ground beetles by two methods: Potentials and methodological pitfalls. Ecology and Evolution, 11(13), 8562-8572. <https://doi.org/10.1002/ece3.7670>
97. Růžičková, J., & Veselý, M. (2018). Movement activity and habitat use of Carabus ullrichii (Coleoptera: Carabidae): The forest edge as a mating site?. Entomological Science, 21(1), 76-83. <https://doi.org/10.1111/ens.12286>
98. Růžičková, J., & Veselý, M. (2016). Using radio telemetry to track ground beetles: Movement of Carabus ullrichii. Biologia, 71(8), 924-930. <https://doi.org/10.1515/biolog-2016-0108>
99. Růžičková, J., Bérces, S., Ackov, S., & Elek, Z. (2021). Individual movement of large carabids as a link for activity density patterns in various forestry treatments. Acta Zoologica Academiae Scientiarum Hungaricae, 67(1), 77-86. <https://doi.org/10.17109/AZH.67.1.77.2021>
100. Rykken, J. J., Jepson, P. C., & Moldenke, A. R. (2011). Ground-dwelling arthropod distribution and movement across a fragmented riparian forest. Northwest Science, 85(4), 527-541. <https://doi.org/10.3955/046.085.0403>
101. Samietz, J., & Berger, U. (1997). Evaluation of movement parameters in insects–bias and robustness with regard to resight numbers. Oecologia, 110(1), 40-49. <https://doi.org/10.1007/s004420050131>
102. Samu, F., & Sárospataki, M. (1995). Estimation of population sizes and" home ranges" of polyphagous predators in alfalfa using mark-recapture: an exploratory study. Acta Jutlandica, 70, 47-56.
103. Skłodowski, J. (2008). Carabid beetle movements in a clear-cut area with retention groups of trees. In: Back to the Roots and Back to the Future (pp. 20-24). Pensoft Publishers. Sofia–Moscow.
104. Skłodowski, J. (1999). Movement of selected carabid species (Col. Carabidae) through a pine forest-fallow ecotone. Folia Forestalia Polonica Series A Forestry, 41, 5-23.
105. Skłodowski, J., & Szczeszek, J. (2015). Dead wood modifies mobility of ground beetles. Baltic Journal of Coleopterology, 15(2), 91-98.
106. Stringer, I. A., & Chappell, R. (2008). Possible rescue from extinction: transfer of a rare New Zealand tusked weta to islands in the Mercury group. In Insect Conservation and Islands (pp. 177-188). Dordrecht: Springer Netherlands. <https://doi.org/10.1007/978-1-4020-8782-0_15>
107. Szyszko, J., Gryuntal, S., & Schwerk, A. (2005). Nocturnal activity of Carabus hortensis L.(Coleoptera, Carabidae) in two forest sites studied with harmonic radar method. Polish Journal of Ecology, 53(1), 117-121.
108. Szyszko, J., Gryuntal, S., & Schwerk, A. (2004). Differences in locomotory activity between male and female Carabus hortensis (Coleoptera: Carabidae) in a pine forest and a beech forest in relation to feeding state. Environmental Entomology, 33(5), 1442-1446. <https://doi.org/10.1603/0046-225X-33.5.1442>
109. Testud, G., Vergnes, A., Cordier, P., Labarraque, D., & Miaud, C. (2019). Automatic detection of small PIT-tagged animals using wildlife crossings. Animal Biotelemetry, 7(1), 21. <https://doi.org/10.1186/s40317-019-0183-5>
110. Thomas, C. F. G., Brown, N. J., & Kendall, D. A. (2006). Carabid movement and vegetation density: Implications for interpreting pitfall trap data from split-field trials. Agriculture, Ecosystems & Environment, 113(1-4), 51-61. <https://doi.org/10.1016/j.agee.2005.08.033>
111. Thomas, C. F. G., Parkinson, L., & Marshall, E. J. P. (1998). Isolating the components of activity-density for the carabid beetle Pterostichus melanarius in farmland. Oecologia, 116(1), 103-112. <https://doi.org/10.1007/s004420050568>
112. Varga, S., & Kenyeres, Z. (2025). Should I stay or should I go? Comparison of movement patterns of a flightless bush-cricket in sparse versus dense grassland. Acta Oecologica, 127, 104072. <https://doi.org/10.1016/j.actao.2025.104072>
113. Vergnes, A., Chantepie, S., Robert, A., & Clergeau, P. (2013). Are urban green spaces suitable for woodland carabids? First insights from a short-term experiment. Journal of insect conservation, 17(4), 671-679. <https://doi.org/10.1007/s10841-013-9551-2>
114. Vermeulen, H. J. (1994). Corridor function of a road verge for dispersal of stenotopic heathland ground beetles Carabidae. Biological Conservation, 69(3), 339-349. <https://doi.org/10.1016/0006-3207(94)90433-2>
115. Vinatier, F., Chailleux, A., Duyck, P. F., Salmon, F., Lescourret, F., & Tixier, P. (2010). Radiotelemetry unravels movements of a walking insect species in heterogeneous environments. Animal behaviour, 80(2), 221-229. <https://doi.org/10.1016/j.anbehav.2010.04.022>
116. Volf, M., Holec, M., Holcová, D., Jaroš, P., Hejda, R., Drag, L., Blízek, J., Šebek, P., & Čížek, L. (2018). Microhabitat mosaics are key to the survival of an endangered ground beetle (Carabus nitens) in its post-industrial refugia. Journal of Insect Conservation, 22(2), 321-328. <https://doi.org/10.1007/s10841-018-0064-x>
117. Vom Hofe, H., & Gerstmeier, R. (2001). Ecological preferences and movement patterns of carabid beetles along a river bank. Revue d'écologie, 56(4), 313-320.
118. Wallin, H. (1991). Movement patterns and foraging tactics of a caterpillar hunter inhabiting alfalfa fields. Functional Ecology, 5(6), 740-749. <https://doi.org/10.2307/2389536>
119. Wallin, H., & Ekbom, B. (1994). Influence of hunger level and prey densities on movement patterns in three species of Pterostichus beetles (Coleoptera: Carabidae). Environmental entomology, 23(5), 1171-1181. <https://doi.org/10.1093/ee/23.5.1171>
120. Wallin, H., & Ekbom, B. S. (1988). Movements of carabid beetles (Coleoptera: Carabidae) inhabiting cereal fields: a field tracing study. Oecologia, 77(1), 39-43. <https://doi.org/10.1007/BF00380922>
121. Walters, R. J., Hassall, M., Telfer, M. G., Hewitt, G. M., & Palutikof, J. P. (2006). Modelling dispersal of a temperate insect in a changing climate. Proceedings of the Royal Society B: Biological Sciences, 273(1597), 2017-2023. <https://doi.org/10.1098/rspb.2006.3542>
122. Watts, C., Empson, R., Thornburrow, D., & Rohan, M. (2012). Movements, behaviour and survival of adult Cook Strait giant weta (Deinacrida rugosa; Anostostomatidae: Orthoptera) immediately after translocation as revealed by radiotracking. Journal of Insect Conservation, 16(5), 763-776. <https://doi.org/10.1007/s10841-012-9461-8>
123. Watts, C., Stringer, I., Thornburrow, D., & MacKenzie, D. (2011). Are footprint tracking tunnels suitable for monitoring giant weta (Orthoptera: Anostostomatidae)? Abundance, distribution and movement in relation to tracking rates. Journal of Insect Conservation, 15(3), 433-443. <https://doi.org/10.1007/s10841-010-9321-3>
124. Wehnert, A., & Wagner, S. (2019). Niche partitioning in carabids: single‐tree admixtures matter. Insect Conservation and Diversity, 12(2), 131-146. <https://doi.org/10.1111/icad.12321>
125. Wehnert, A., Wagner, S., & Huth, F. (2020). Spatio-Temporal Distribution of Carabids Influenced by Small-Scale Admixture of Oak Trees in Pine Stands. Diversity, 12(10), 398. <https://doi.org/10.3390/d12100398>
126. Weyer, J., Weinberger, J., & Hochkirch, A. (2012). Mobility and microhabitat utilization in a flightless wetland grasshopper, Chorthippus montanus (Charpentier, 1825). Journal of Insect Conservation, 16(3), 379-390. <https://doi.org/10.1007/s10841-011-9423-6>
127. Wiens, J. A., & Milne, B. T. (1989). Scaling of ‘landscapes’ in landscape ecology, or, landscape ecology from a beetle's perspective. Landscape ecology, 3(2), 87-96. <https://doi.org/10.1007/BF00131172>
128. Wiens, J. A., Crist, T. O., & Milne, B. T. (1993). On quantifying insect movements. Environmental Entomology, 22(4), 709-715. <https://doi.org/10.1093/ee/22.4.709>
129. With, K. A. (1994). Ontogenetic shifts in how grasshoppers interact with landscape structure: an analysis of movement patterns. Functional Ecology, 8(4), 477-485. <https://doi.org/10.2307/2390072>
130. Yagui, H., Kearney, M. R., & Hoffmann, A. A. (2024). Restoring declining species through translocations: A test case using flightless grasshoppers in an urban setting. Insect Conservation and Diversity, 17(2), 358-373. <https://doi.org/10.1111/icad.12679>
131. Yamada, Y., Sasaki, H., & Harauchi, Y. (2010). Effects of narrow roads on the movement of carabid beetles (Coleoptera, Carabidae) in Nopporo Forest Park, Hokkaido. Journal of Insect Conservation, 14(2), 151-157. <https://doi.org/10.1007/s10841-009-9236-z>
132. Zhang, J., Drummond, F. A., Liebman, M., & Hartke, A. (1997). Phenology and dispersal of Harpalus rufipes DeGeer(Coleoptera: Carabidae) in agroecosystems in Maine. Journal of Agricultural Entomology, 14(2), 171-186.
